# Hierarchical organization and genetically separable subfamilies of PSD95 postsynaptic supercomplexes

**DOI:** 10.1111/jnc.14056

**Published:** 2017-07-25

**Authors:** René A. W. Frank, Fei Zhu, Noboru H Komiyama, Seth G. N. Grant

**Affiliations:** ^1^ MRC Laboratory of Molecular Biology Francis Crick Avenue Cambridge UK; ^2^ Centre for Clinical Brain Sciences University of Edinburgh Chancellor's Building Little France Crescent Edinburgh UK; ^3^ Present address: Institute of Neurology University College London Queen Square, London UK

**Keywords:** Ion channel supercomplexes, Kir2.3, NMDA receptor, PSD95, synapse diversity, synapse proteome

## Abstract

PSD95 is an abundant postsynaptic scaffold protein in glutamatergic synapses that assembles into supercomplexes composed of over 80 proteins including neurotransmitter receptors, ion channels and adhesion proteins. How these diverse constituents are organized into PSD95 supercomplexes *in vivo* is poorly understood. Here, we dissected the supercomplexes in mice combining endogenous gene‐tagging, targeted mutations and quantitative biochemical assays. Generating compound heterozygous mice with two different gene‐tags, one on each *Psd95* allele, showed that each ~1.5 MDa PSD95‐containing supercomplex contains on average two PSD95 molecules. Gene‐tagging the endogenous GluN1 and PSD95 with identical Flag tags revealed N‐methyl D‐aspartic acid receptors (NMDARs) containing supercomplexes that represent only 3% of the total population of PSD95 supercomplexes, suggesting there are many other subtypes. To determine whether this extended population of different PSD95 supercomplexes use genetically defined mechanisms to specify their assembly, we tested the effect of five targeted mouse mutations on the assembly of known PSD95 interactors, Kir2.3, Arc, IQsec2/BRAG1 and Adam22. Unexpectedly, some mutations were highly selective, whereas others caused widespread disruption, indicating that PSD95 interacting proteins are organized hierarchically into distinct subfamilies of ~1.5 MDa supercomplexes, including a subpopulation of Kir2.3‐NMDAR ion channel‐channel supercomplexes. Kir2.3‐NMDAR ion channel‐channel supercomplexes were found to be anatomically restricted to particular brain regions. These data provide new insight into the mechanisms that govern the constituents of postsynaptic supercomplexes and the diversity of synapse types.

**Read the Editorial Highlight for this article on** page 500.

**Cover Image for this issue: doi.** 10.1111/jnc.13811.

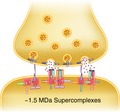

**Abbreviations used**
0.8‐NR. 0.8 MDa: NMDAR channel complex
1.5‐Adam22: ˜1.5 MDa Adam22‐containing supercomplex
1.5‐Arc: ˜1.5 MDa Arc‐containing supercomplex
1.5‐IQsec2: ˜1.5 MDa IQsec2/BRAG1‐containing supercomplex
1.5‐Kir2.3: ˜1.5 MDa Kir2.3‐containing supercomplex
1.5‐NR: ˜1.5 NMDAR‐containing supercomplex
1.5‐PSD95: ˜1.5 MDa PSD95‐containing supercomplex
NMDAR: N‐methyl D‐aspartic acid receptors
PSD95‐GFP: C‐terminal GFP‐tagged PSD95
TAP: tandem‐affinity purification
WT: wildtype

PSD95 is a scaffold protein composed of three PDZ (PSD95, Dlg, ZO‐1 homologous region) domains, an SH3 domain and a guanylate kinase domain that mediate interactions with numerous synaptic proteins including neurotransmitter receptors, adhesion and signalling proteins (Husi *et al*. 2000; Fernández *et al*. 2009; Zhu *et al*. 2016). However, the size, stoichiometry and in what quaternary molecular state PSD95 is assembled *in vivo* is presently unclear (Hsueh *et al*. 1997; Christopherson *et al*. 2003; Zeng *et al*. 2016). We recently reported that PSD95 resides almost exclusively within ~1.5 MDa supercomplexes and that NMDARs are organized into supercomplexes containing both PSD95 and PSD93 (Frank *et al*. 2016). Here, using an integrated genetic and biochemical strategy, we show that, on average, a dimer of PSD95 hierarchically organizes postsynaptic proteins into multiple distinct ~1.5 MDa PSD95 supercomplex subfamilies in the brain.

## Materials and methods

### Antibodies

The following antibodies were used in this study: mAb Flag [F3165, (RRID:AB_259529); Sigma, St Louis, MO, USA], mAb Flag‐HRP [A8592 (RRID:AB_439702); Sigma], mAb GFP [A11120 (RRID:AB_221568); Thermo Fisher Scientific Inc., Waltham, MA, USA], mAb PSD95 [MA1‐045 (RRID:AB_325399); Thermo], mAb PSD93 [Neuromab, 75‐057 (RRID:AB_2277296)], mAb GluN1 [32‐0500 (RRID:AB_2533060); Thermo], pAb IQsec2 (gift from Professor James Casanova, University of Virginia, RRID:AB_2636960)], mAb Adam22 [Neuromab, 75‐083 (RRID:AB_10675128)], pAb Arc [Synaptic systems, 156003 (RRID:AB_887694)], mAb Kir2.3 [Neuromab, 75‐069 (RRID:AB_2130742)].

### Animals

All animal experiments conformed to the British Home Office Regulations (Animal Scientific Procedures Act 1986; Project License PPL80/2337 to Seth Grant), local ethical approval and NIH guidelines. All mutant mice were made by homologous recombination in embryonic stem cells. The generation of *Glun1* ^*TAP*^ (Frank *et al*. 2016), *Psd95* ^*TAP*^ (Fernández *et al*. 2009), *Psd95* ^*−/−*^ (Migaud *et al*. 1998), *Psd95* ^*EGFP*^ (Broadhead *et al*. 2016), *Psd93* ^*−/−*^ (McGee *et al*. 2001), *Glun2b* ^*2A(CTR)*^, *Glun2a* ^*2B(CTR)*^ (Ryan *et al*. 2013) and *Glun2a* ^*del‐CTD*^ (gift of P. H. Seeburg and R. Sprengel) (Sprengel *et al*. 1998) strains of mice were previously reported. Since the proteins under investigation showed no sex dimorphism, male and female mice were used for biochemical assays.

### Blue native and SDS‐PAGE immunoblot

Adult (P56‐70) mouse forebrains (cortex and hippocampus) were dissected and homogenized in buffer H (1 mM Na HEPES pH7.4, 320 mM sucrose with protease inhibitors). Samples were collected for sodium dodecyl sulfate–polyacrylamide gel electrophoresis (SDS‐PAGE). The homogenate pellet was collected by centrifugation with 1168 g. (MLA‐80, 5000 rpm) at 2°C for 10 min and re‐homogenized (6 strokes) in 2 mL buffer H and centrifuged as before. Pooled first and second 1168 g. supernatants were centrifuged at 16860 g. (MLA80, 19 000 rpm) to pellet the crude membranes. Crude membranes were re‐suspended in 2.5 mL buffer H and extracted with 2.5 mL buffer X (1% Na deoxycholate, 100 mM NaCl, 50 mM tris.Cl pH8) for 1 h at 6–10°C. Next, insoluble and non‐specifically aggregated proteins were removed from the total extract by centrifugation at 116760 g. (MLA‐80, 50 000 rpm) for 40 min at 8°C. Samples were collected for blue native PAGE (BNP) and immediately run according to Schagger (Schagger 2001) followed by immunoblot.

### Immunohistochemistry

Immunohistochemistry of homozygous PSD95 knockout (*Psd95* ^*−/−*^) and wildtype mice was carried out as recently described (Frank *et al*. 2016). Kir2.3 (Neuromab, 75‐069) and synapsin 1 (Thermofisher, OPA1‐04001) were both used at 1 : 200 dilution.

### Brain region‐specific purification of TAP‐tagged GluN1 supercomplexes

Adult (P56‐P150) *Glun1* ^*TAP/TAP*^ mouse forebrains were collected and immediately cut into 400 μm coronal sections using a McIlwain ‘tissue chopper’. Two anatomical samples were collected and flash frozen: (i) striatum including the cordate putamen, olfactory tubercle and piriform cortex, and (ii) the posterior cortex and hippocampus. About 240‐320 mg tissue from 3 to 5 animals was used in each purification. The volumes of all buffers were scaled to the brain tissue weight as indicated below. Samples were homogenized (12 strokes with a Teflon‐glass pestle and mortar) in 21.5 μL/mg buffer H (1 mM Na HEPES pH7.4, 320 mM sucrose with protease inhibitors). The homogenate pellet was collected by centrifugation with 1168 *g*. (MLA‐80, 5000 rpm) at 2°C for 10 min and re‐homogenized (6 strokes) in 8 μL/mg buffer H. The first and second 1168 g. supernatants were pooled and centrifuged at 16860 *g*. (MLA80, 19 000 rpm) to pellet the crude synaptoneurosome membranes. The pellet was re‐suspended in 10 μL/mg buffer H and extracted with 10 μL/mg 2% deoxycholate, 100 mM NaCl, 50 mM Tris.Cl pH8 for 1 h at 6°C. Total extract was centrifuged at 120 000 g. for 40 min at 8°C; 70 μg/mg mouse Flag antibody was coupled to 33 μg/mg protein G magnetic beads (Invitrogen). Receptor was captured from extract supernatant for 2 h. The beads were washed three times with 5 μL/mg wash buffer (0.37% w/v sodium deoxycholate, 0.05 mg/mL lipids [1 : 1 : 3 POPC:POPE:POG (1‐palmitoyl‐2‐oleoyl‐*sn*‐glycero‐3‐phosphocholine, 1‐palmitoyl‐2‐oleoyl‐*sn*‐glycero‐3‐phosphoethanolamine, 1‐palmitoyl‐2‐oleoyl‐*sn*‐glycero‐3‐phospho‐(1′‐*rac*‐glycerol)], 150 mM NaCl, 50 mM Tris.Cl pH8). Flag captured complexes were eluted with 2.6 μL/mg wash buffer supplemented with 0.2 mg/mL Flag peptide for 2 h at 6°C. Eluate was buffer exchanged and concentrated with a 100‐kDa MWCO (molecular weight cutt off) centrifugal filter (Merck, Darmstadt, Germany) to 20 μL for BNP immunoblot.

### Statistical analysis

All experiments were performed using at least six biological and two technical replicates. Student's *t*‐test was used to compare two experimental groups. *p* values < 0.05 were considered as statistically significant.

## Results

### Using targeted genetic tags to quantify 1:17 molar ratio of NMDAR to PSD95 in the mouse forebrain

**Figure 1:**
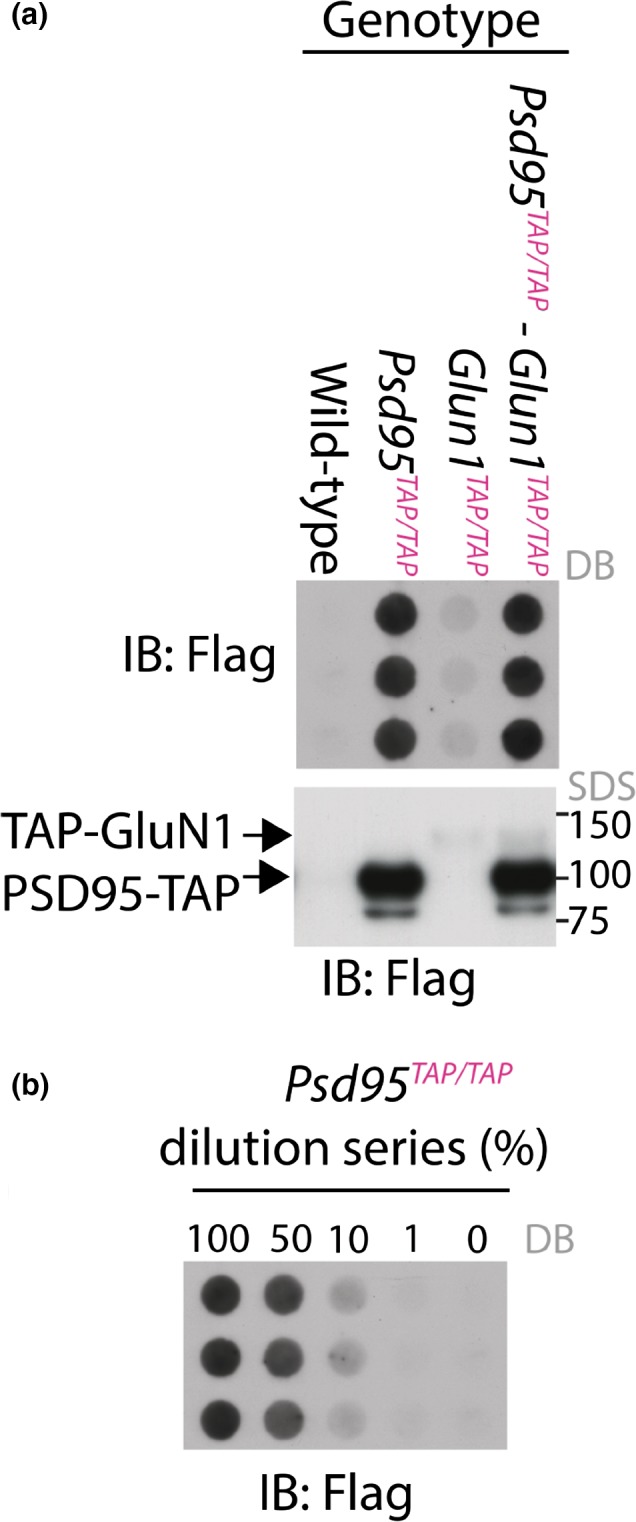
Quantification of the GluN1/PSD95 relative abundance *in vivo* gave a 17 : 1 molar ratio of PSD95 over GluN1. (a) GluN1 and PSD95 were detected by sulfate–polyacrylamide gel electrophoresis (SDS‐PAGE) (lower panel) and triplicate dot blot (DB) Flag immunoblot (upper panel) of *Glun1* ^*TAP*^ ^*/*^ ^*TAP*^,* Psd95* ^*TAP*^ ^*/*^ ^*TAP*^ mouse forebrains respectively. The TAP‐GluN1 and PSD95‐TAP SDS‐PAGE bands are similar to their counterparts in singularly TAP‐tagged mice (*Psd95* ^*TAP*^ ^*/*^ ^*TAP*^ and *Glun1* ^*TAP*^ ^*/*^ ^*TAP*^), which indicates equal loading. (b) The molar ratio of Flag from *Psd95* ^*TAP*^ ^*/*^ ^*TAP*^ and *Glun1* ^*TAP*^ ^*/*^ ^*TAP*^ was quantified densitometrically using a dilution series, in which *Psd95* ^*TAP*^ ^*/*^ ^*TAP*^ forebrain extracts were diluted with that of wildtype. Densitometry of dilution series indicated TAP‐PSD95 was 17 ± 3‐fold (mean ± SD) more concentrated than TAP‐GluN1.

We recently reported that NMDARs were partitioned between ~0.8 MDa tetrameric ion channel complexes and ~1.5 MDa supercomplexes (hereafter referred to as 0.8‐NR and 1.5‐NR respectively), whereas almost all forebrain PSD95 was retained within ~1.5 MDa supercomplexes (hereafter referred to as 1.5‐PSD95) (Frank *et al*. 2016). To quantify the molar ratio of PSD95 and NMDARs, we used two knockin mouse lines where the PSD95 and the obligatory subunit of NMDARs, GluN1, were tagged with an identical 3xFlag tag targeted to the genes encoding these proteins (*Glun1* ^*TAP/TAP*^ and *Psd95* ^*TAP/TAP*^, respectively) (Frank *et al*. 2016). In dotblots and SDS‐PAGE immunoblots, no Flag was detected in wildtype mouse total forebrain, whereas that in *Glun1* ^*TAP/TAP*^ and *Psd95* ^*TAP/TAP*^ mice indicated the amount of PSD95 and GluN1, respectively (Fig. 1a). The intensity measured by densitometry of immuno‐dotblots from *Glun1* ^*TAP/TAP*^ mouse forebrains is 6 ± 1% that of *Psd95* ^*TAP/TAP*^, which corresponds to a 17 ± 3 fold (mean ± SD) molar excess of PSD95 over GluN1 (Fig. 1a and b). Since ~50% of GluN1 subunits are assembled with PSD95 (Frank *et al*. 2016), 1.5‐NRs represent a ~3% subset of an extended family of ~1.5 MDa supercomplexes containing PSD95.

### Using mouse genetics to measure the oligomeric state of PSD95 in ~1.5 MDa supercomplexes

**Figure 2:**
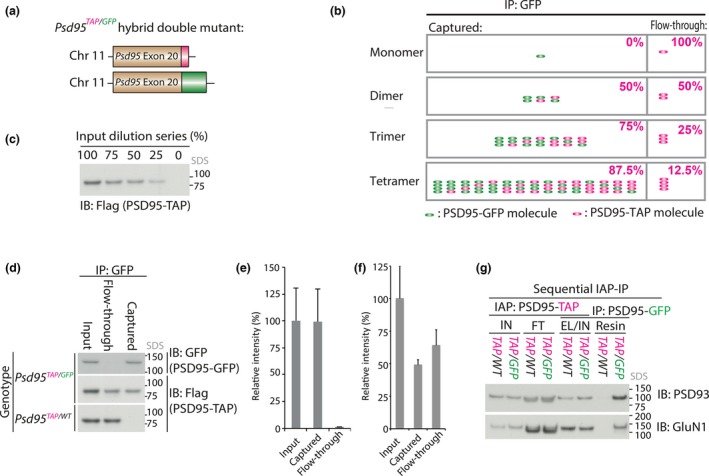
Quantification of the *in vivo* stoichiometry of PSD95 in each 1.5‐PSD95 using compound heterozygous knockin tags (*Psd95* ^*TAP*^ ^*/*^ ^*GFP*^). (a) Schematic showing the gene structure of the last protein coding exon (exon 20) of *Psd95* in *Psd95* ^*TAP*^ ^*/*^ ^*GFP*^ hybrid mutant mice. Equal expression of both alleles is expected in each cell because *Psd95* is on an autosomal chromosome (Chr 11). (b) Schematic showing the expected partitioning of TAP‐tagged PSD95 between captured (resin) and flow‐through (unbound) in a GFP immunoprecipitation (IP) from *Psd95* ^*TAP*^ ^*/*^ ^*GFP*^ hybrid mutant forebrain extract. Only PSD95‐TAP assemblies containing at least one PSD95‐GFP will be captured and the expected distribution of PSD95‐TAP and PSD95‐GFP subunits between captured and flow‐through samples is depicted for different homo‐oligomeric states of 1.5‐PSD95 containing on average: 1 (monomer), 2 (dimer), 3 (trimer) or 4 (tetramer) PSD95 molecules. This partitioning, indicated as the percentage split for PSD95‐TAP captured and in the flow‐through, is dependent on the stoichiometry of PSD95 molecules in each complex. Green and cyan ellipses correspond to PSD95‐GFP and PSD95‐TAP subunits, respectively. For each oligomeric state, all possible assemblies containing different combinations of GFP‐ and TAP‐tagged PDS95 are shown. (c) Dilution series of *Psd95* ^*TAP*^ ^*/*^ ^*GFP*^ forebrain extract into that of wildtype indicated sensitivity of quantification. These data show the dynamic range of sulfate–polyacrylamide gel electrophoresis (SDS‐PAGE) Flag immunoblot detection. (d) PSD95‐GFP was immunoprecipitated (IP) from *Psd95* ^*TAP*^ ^*/*^ ^*GFP*^ and negative control (*Psd95* ^*TAP*^ ^*/*^ ^*WT*^) forebrain extract supernatant (Input) with GFP antibody. Top panel, SDS‐PAGE GFP immunoblot of IP shows near complete immunoprecipitation of all PSD95‐GFP (Captured) from *Psd95* ^*TAP*^ ^*/*^ ^*GFP*^ hybrid double mutant forebrain extracts. Second panel, Flag immunoblot shows half PSD95‐TAP co‐precipitated with PSD95‐GFP, the remaining unbound PSD95‐TAP was detected in the flow‐through. Lower panel, Flag immunoblot of control IP (*Psd95* ^*TAP*^ ^*/*^ ^*WT*^) shows no PSD95‐TAP was captured in the absence of GFP‐tagged PSD95. The total protein loaded for SDS immunoblot was normalized across *Input*,* Flow‐through* and *Captured* lanes by supplementing with non‐tagged (wildtype)samples. Representative data from triplicate experiments shown. (e) Densitometric immunoblot quantification of PSD95‐GFP (GFP) in GFP IP. The band intensities from triplicate samples were measured and normalized to that of input. Error bars indicate 1 SD. These data show essentially all the PSD95‐GFP was captured by GFP IP. (f) Densitometric immunoblot quantification of PSD95‐TAP (Flag) in GFP IP (g, middle panel). The band intensities from triplicate samples were measured and normalized to that of input. Error bars indicate 1 SD. These data show half the PSD95‐TAP was co‐captured by PSD95‐GFP immunoprecipitation. Thus, each 1.5‐PSD95 supercomplex contains on average a dimer of PSD95 molecules. (g) 1.5‐PSD95 each containing two molecules of PSD95 were isolated in two sequential steps: Flag immunoaffinity purification (‘IAP’) followed by GFP immunoprecipitation (‘IP’) from *Psd95* ^*TAP/GFP*^ hybrid double mutant mice. *Psd95* ^*TAP/WT*^ mice were used as a negative control. PSD93 and GluN1 were detected by SDS‐PAGE immunoblot. These data show that the subset of 1.5‐PSD95 containing PSD93 and GluN1 each also contain a dimer of PSD95.

It is possible that multiple copies or oligomers of PSD95 are found in each 1.5‐PSD95 (Hsueh *et al*. 1997; Christopherson *et al*. 2003; Zhu *et al*. 2016). To measure the average number of PSD95 molecules in each ~1.5 MDa supercomplex in the mouse forebrain, we targeted two different tags, 3xFlag (Fernández *et al*. 2009) and GFP (Broadhead *et al*. 2016); one to each allele of the gene encoding PSD95 (*Psd95*) to produce a compound heterozygous knockin line, *Psd95* ^*TAP/EGFP*^ (Fig. 2a). Since equal expression of both alleles is expected in each cell, observing the ratio of co‐assembly of PSD95‐TAP and PSD95‐GFP in forebrain extracts gave a direct measure of the average number of PSD95 molecules in each complex. If only a single molecule of PSD95 were required in each complex, immuno‐capture of PSD95‐GFP with anti‐GFP antibody would co‐purify none of the TAP‐tagged PSD95. If on average two, three or four molecules of PSD95 assemble in each complex, 50%, 75% or 87.5% PSD95‐TAP would be co‐captured with PSD95‐GFP respectively (Fig. 2b). As seen in Fig. 2c–f when all PSD95‐GFP was immuno‐captured, 49 ± 3% (mean ± SD) PSD95‐TAP was co‐purified, indicating that each complex contains on average two molecules of PSD95.

~1.5 MDa NMDAR supercomplexes contain both PSD95 and PSD93 (Frank *et al*. 2016). In accordance, serial purification of 3×Flag and GFP from *Psd95* ^*TAP/EGFP*^ compound heterozygous mice showed that dimers of PSD95 also contain NMDAR and PSD93 (Fig 2f). The mass of a dimer of PSD95 is ~170 kDa, thus the remaining mass of 1.5‐PSD95 must be occupied by other proteins.

### Genetic and biochemical dissection of NMDAR and PSD95 supercomplex subfamilies

**Figure 3:**
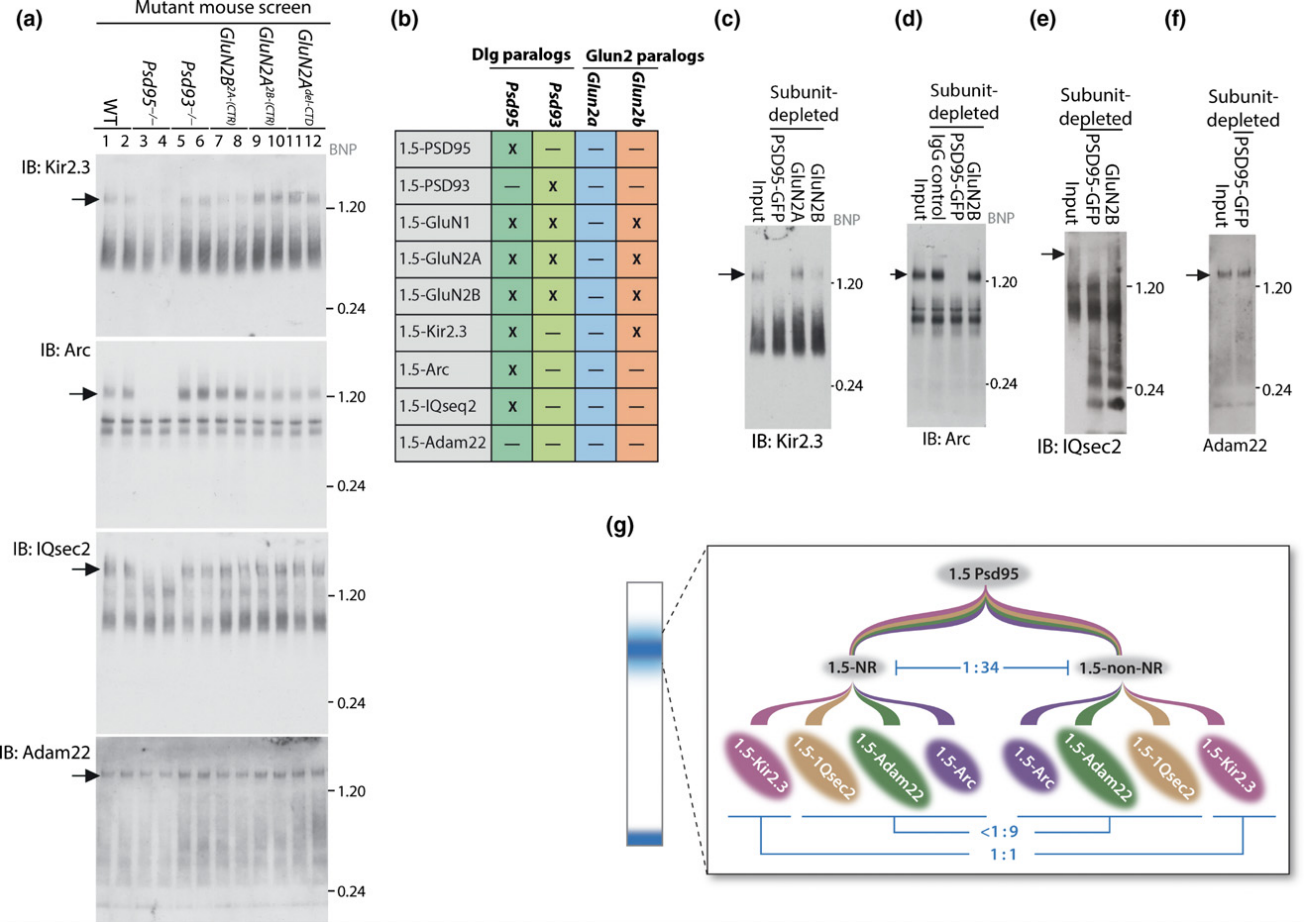
Characterization of supercomplex subtypes. (a) Mutant mouse screen of synaptic supercomplexes. BNP immunoblots of forebrain extracts from five mutant mouse lines show genetic dependencies for the assembly of 1.5‐Kir2.3, 1.5‐Arc, 1.5‐IQsec2 and 1.5‐Adam22. Each panel contains duplicates of wildtype (WT) (lane 1,2, duplicates), *Psd95* ^*−/−*^ (lane 3,4), *Psd93* ^*−/−*^ (lane 5,6), *Glun2b* ^*2A(*^ ^*CTR*^ ^*)/2A(*^ ^*CTR*^ ^*)*^ (lane 7,8), *Glun2a* ^*2B(*^ ^*CTR*^ ^*)/2B(*^ ^*CTR*^ ^*)*^ (lane 9,10), *GluN2a* ^*del*^ ^*CTD*^. Immunoblotting antibody is indicated below each panel (IB). Arrow indicates 1.5 MDa bands. Molecular weight in MDa shown on right. Representative results from triplicate experiments shown. (b) Table summarizing data in Fig. 3a and recently published (Frank *et al*. 2016) showing the effect of different mutations (columns) on distinct components of 1.5‐PSD95 supercomplexes (rows). X, denotes assembly of supercomplex was blocked by the mutation. ‐, denotes assembly of the supercomplex was not blocked by the mutation. (c) Subunit‐depletion of GluN2B and PSD95 removes 1.5‐Kir2.3. Extracts from *Glun1* ^*TAP*^ ^*/*^ ^*TAP*^ */Psd95* ^*GFP*^ ^*/*^ ^*GFP*^ double knockin mice were subunit‐depleted with antibodies (shown in lanes), then separated on BNP for immunoblotting with Kir2.3 antibody to show 1.5‐Kir2.3. Lanes; Input, total extract; immunodepleting antibodies (lanes shown left to right), non‐specific IgG, GFP, GluN2A, GluN2B. Arrow indicates 1.5‐Kir2.3. Molecular weight in MDa shown on right. IB, immunoblotting antibody. (d) Subunit‐depletion of PSD95 removes all 1.5‐Arc. Extracts from *Glun1* ^*TAP*^ ^*/*^ ^*TAP*^ */Psd95* ^*GFP*^ ^*/*^ ^*GFP*^ double knockin mice were subunit‐depleted with antibodies (shown in lanes) then separated on BNP for immunoblotting with Arc antibody to show 1.5‐Arc. Lanes; Input, total extract; immunodepleting antibodies (lanes shown left to right), non‐specific IgG, GFP, GluN2B. Arrow indicates 1.5‐Arc. Molecular weight in MDa shown on right. IB, immunoblotting antibody. (e) Subunit‐depletion of PSD95 removes all 1.5‐IQsec. Extracts from *Glun1* ^*TAP*^ ^*/*^ ^*TAP*^ */Psd95* ^*GFP*^ ^*/*^ ^*GFP*^ double knockin mice were subunit‐depleted with antibodies (shown in lanes), then separated on BNP for immunoblotting with IQsec2 antibody to show 1.5‐IQsec2. (f) Subunit‐depletion of PSD95 does not remove all Adam22. Extracts from *Glun1* ^*TAP*^ ^*/*^ ^*TAP*^ */Psd95* ^*GFP*^ ^*/*^ ^*GFP*^ double knockin mice were subunit‐depleted with antibodies (shown in lanes) then separated on BNP for immunoblotting with Adam22 antibody to show 1.5‐Adam22. (g) Schematic showing extended family tree of ~1.5 MDa supercomplexes that contain PSD95 and their relative abundance in the mouse forebrain. The 1.5‐PSD95 was divided into 1.5‐NR and 1.5‐Non‐NR subpopulations. PSD95 is 17‐fold more abundant than GluN1. Since ~50% N‐methyl D‐aspartic acid receptors (NMDARs) interact with PSD95 (Frank *et al*. 2016), 1.5‐Non‐NR is 34‐fold more abundant than 1.5‐NR (ratio indicated in blue). Each subpopulation was further subdivided into those containing Kir2.3, IQseq2, Adam22 and Arc. The distribution of 1.5‐Kir2.3, 1.5‐IQsec2, 1.5‐Adam22 and 1.5‐Arc (expressed as a ratio in blue) between 1.5‐NR and 1.5‐Non‐NR were estimated by densitometry of supercomplexes immunodepleted with GluN2B and PSD95‐EGFP respectively (see Fig. 3c–e).

Mass spectrometric analysis of 1.5‐NR and 1.5‐PSD95 supercomplexes identified 55 and 79 different proteins, respectively (Fernández *et al*. 2009; Frank *et al*. 2016), with 89% identity. To determine whether the constituents of 1.5‐NR and 1.5‐PSD95 are in overlapping or separable populations of supercomplexes, we focussed on four constituents of 1.5‐NR and 1.5‐PSD95 that were readily detected by blue native PAGE (BNP) immunoblot in wildtype forebrain extract (Frank *et al*. 2016): the inward‐rectifying potassium channel, Kir2.3, an ARF‐GEF signalling cofactor, IQsec2/Brag1, an immediate early gene product, Arc/Arg3.1, and a trans‐synaptic adhesion protein, Adam22 (Fig. 3a, left most two lanes). Immunoblots show each of these proteins were partitioned into multiple distinct assemblies that all included a discrete band migrating with masses that ranged from 1.2 to 3 MDa, hereafter referred to for simplicity as 1.5‐Kir2.3, 1.5‐IQsec2, 1.5‐Arc, and 1.5‐Adam22, respectively. We next examined these protein complexes in a battery of mutant mice to identify common and distinct genetic requirements of supercomplex assembly.

Since we had previously demonstrated that the 1.5‐NR supercomplex is disrupted in *Psd95* ^*−/−*^ *, Psd93* ^*−/−*^ and *Glun2b* ^*2A(CTR)/2A(CTR)*^ mice (Frank *et al*. 2016), we tested whether these mutants and two other NMDAR mutants (*Glun2a* ^*2B(CTR)/2B(CTR)*^, *Glun2a* ^*del‐CTD*^) disrupted 1.5‐Kir2.3, 1.5‐Arc, 1.5‐IQsec2 and 1.5‐Adam22. Strikingly, as with 1.5‐NR, 1.5‐Kir2.3, 1.5‐Arc and 1.5‐IQsec2 supercomplexes were each totally dependent on PSD95, but unlike 1.5‐NR, they were not totally dependent on PSD93 or GluN2B CTDs (Fig. 3a). Thus, 1.5‐NRs were genetically distinct from 1.5‐Kir2.3, 1.5‐Arc and 1.5‐IQsec2. Moreover, most 1.5‐Adam22 was not affected by any of the mutations, indicating that it was genetically distinct from all the other supercomplexes. These data, summarized in a table (Fig. 3b), show there is a ‘matrix of selectivity’ of mutations for different supercomplexes where some mutations affect multiple supercomplexes (e.g. *Psd95*), whereas other mutations have no effect (GluN2A CTD). Together these results are consistent with the interpretation that distinct PSD95 supercomplexes, each containing combinations of functionally distinct proteins (ion channels, adhesion protein, signalling enzymes), have specific genetic requirements for their assembly.

The genetic experiments revealed further unexpected evidence of subfamilies of PSD95 supercomplexes: we observed a selective loss of half the population of 1.5‐Kir2.3 in *Glun2b* ^*2A(CTR)/2A(CTR)*^ mice (Fig. 3a, lanes 7 and 8), whereas all 1.5‐Kir2.3 were missing in *Psd95* ^*−/−*^ mice (Fig. 3a, lanes 3 and 4). This raises the possibility that there are two genetically separable populations of 1.5‐Kir2.3: those that were assembled with 1.5‐NR into NMDAR‐Kir2.3 ion channel‐channel supercomplexes and those without NMDARs (1.5‐Non‐NR). To test the possibility that there is a subpopulation of 1.5‐Kir2.3 containing NMDARs and another lacking NMDARs, we examined extracts after subunit‐depletion of either NMDARs or PSD95. Immunodepleting PSD95 removed all 1.5‐Kir2.3, whereas immunodepleting GluN2B removed 50% of the population of 1.5‐Kir2.3 (Fig. 3c). These results are consistent with genetic findings suggesting that half the population of 1.5‐Kir2.3 interact with NMDARs within an ion channel‐channel supercomplex, while all other 1.5‐Kir2.3 are in supercomplexes that lack NMDARs. Immunodepleting GluN2A removed almost no 1.5‐Kir2.3 (Fig. 3c), indicating that these supercomplexes are likely composed of GluN1‐GluN2B di‐heteromers.

As shown in Fig. 3g, these findings show a hierarchy of organization of complexes into supercomplexes, in which 1.5‐PSD95 can be divided into a Kir2.3‐containing subpopulations: those with NMDARs (1.5‐NR) and those lacking receptors (1.5‐non‐NR). To ask whether a similar organization applies to other PSD95 interacting proteins, we quantified the amount of Arc and IQsec2 in 1.5‐NR and 1.5‐non‐NR using the immunodepletion strategy. Densitometric quantification of 1.5‐Arc and 1.5‐IQsec2 BNP immunoblot bands from GluN2B and PSD95 immunodepleted samples indicated that all interact with PSD95 but that 4% and 5% contain NMDARs, respectively (Fig. 3d and e). In contrast, only 14% of 1.5‐Adam22 was removed (Fig. 3f) from PSD95‐depleted samples, suggesting that only a very small fraction of 1.5‐Adam22 contains PSD95, again consistent with genetic findings (Fig. 3a). Densitometric quantification of these data are summarized Fig. 3g (blue annotation) showing the quantitative distribution of supercomplex subtypes.

### Purification of NMDAR‐Kir2.3 ion channel‐channel supercomplex from the midbrain of TAP‐tagged knockin mice

**Figure 4:**
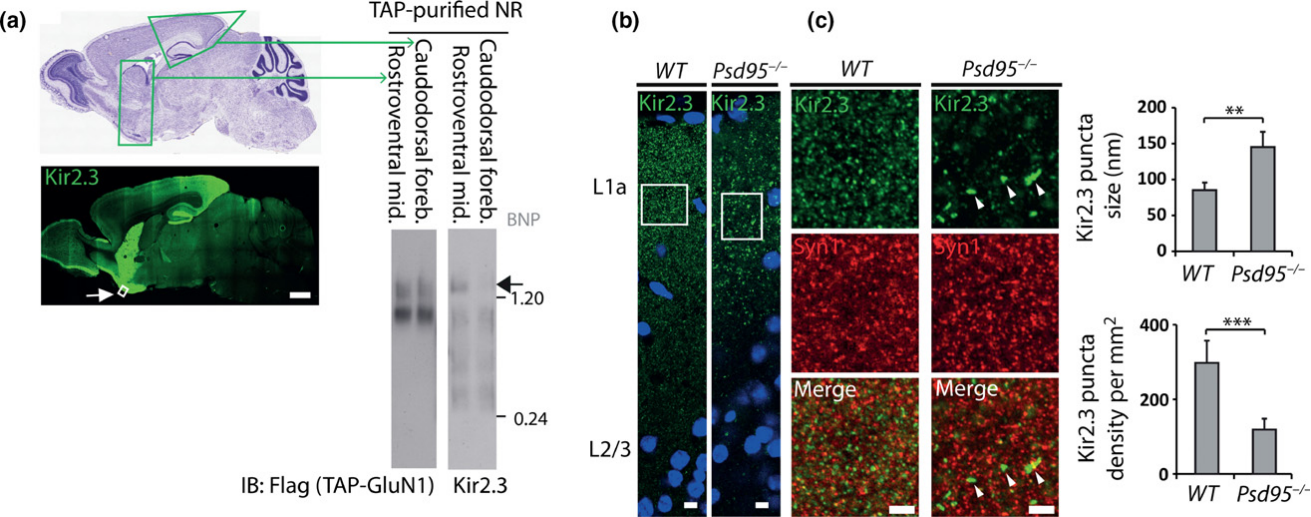
Neuroanatomically restricted assembly of Kir2.3‐N‐methyl D‐aspartic acid receptors (NMDAR) ion channel‐channel supercomplexes. (a) Regional enrichment of Kir2.3 expression in mouse brain. Left, mouse brain sagittal sections (lower) stained with Kir2.3 antibodies and (upper) reference Nissl stain (Allen Brain Atlas). High expression in rostroventral midbrain and caudodorsal forebrain shown and boxed regions were dissected for TAP‐purification of NMDAR. White arrow indicates boxed region of piriform cortex used in high‐magnification images in Fig. 4b and c. Scale bar, 1 mm. Right, BNP immunoblots of TAP‐purified receptors from dissected brain regions of *Glun1* ^*TAP*^ ^*/*^ ^*TAP*^ mice as indicated. Left immunoblot probed with Flag (TAP‐GluN1) and right immunoblot probed with Kir2.3 showing 1.5‐Kir2.3‐NMDAR ion channel‐channel supercomplex enriched in rostroventral midbrain. Filled arrow, 1.5‐Kir2.3/NR. Molecular weight in MDa shown on right. IB, immunoblotting antibody. (b) Kir2.3 localization in layers of piriform cortex of wildtype (WT) and *Psd95* ^*−/−*^ mice, including layers 1a (L1a) and 2/3 (L2/3) of piriform cortex stained with antibodies to Kir2.3 (green) and nuclear stain (DAPI, blue). White boxes indicate regions further magnified in Fig. 4c. Scale bar, 6 μm. (c) Kir2.3 localization requires *Psd95*. Higher magnification of boxed regions in Fig. 4b shows synaptic localization of Kir2.3 is disrupted in Psd95^*−/−*^ mice. Sections double‐stained with Kir2.3 (green, top) and pre‐synaptic marker synapsin1 (red, middle) antibodies and merged image (bottom). White arrowheads show large Kir2.3 aggregates in *Psd95* ^*−/−*^ mice. Scale bar, 4 μm. Right, histograms quantifying changes in puncta size (upper graph) and density (lower graph) of piriform cortex Kir2.3 quantified from triplicate experiments of *Psd95* ^*−/−*^ and WT sections. Error bar, 1 SD. **p ≤* *0.01; ***p ≤* *0.001.

The genetic requirements for supercomplex subtypes could specify assembly anatomically within particular brain regions. To explore whether supercomplexes reside in particular synapses, we used an immunohistochemical survey of Kir2.3 to identify several brain regions enriched in Kir2.3, from which we could isolate Kir2.3‐NMDAR channel‐channel supercomplexes by TAP‐purification using *GluN1* ^*TAP/TAP*^ mice. Kir2.3 resides in postsynaptic puncta enriched in rostroventral midbrain and caudodorsal forebrain (Fig. 4a, left). TAP‐GluN1 purification from isolated brain regions showed 1.5‐Kir2.3‐NMDAR ion channel‐channel supercomplexes were enriched in rostroventral midbrain compared to the caudodorsal forebrain (Fig. 4, right). In accordance with the disruption of 1.5‐Kir2.3‐NMDAR supercomplexes in *Psd95* ^*−/−*^ mice (Fig. 3a), Kir2.3 puncta were selectively disrupted in the rostroventral midbrain of this mutant (Fig. 4b and c). No anatomical difference between *Psd95* ^*−/−*^ and wildtype caudodorsal forebrains was detected.

## Discussion

PSD95 is a central component of the postsynaptic terminal of excitatory synapses with important roles in physiology and behaviour. Although it is known to interact with many proteins and form multiprotein complexes, the stoichiometry of subunits and the specific protein interactions that assemble these complexes is poorly understood in the intact animal. We showed that almost all PSD95 resides within ~1.5 MDa supercomplexes that on average each contain two molecules of PSD95 *in vivo*. Our genetic and biochemical dissection of PSD95 and its interactors suggest that subfamilies of synaptic PSD95 supercomplexes are organized according to a combination of genetic requirements that hierarchically specify their composition.

Although NMDAR and PSD95 both co‐exist within ~1.5 MDa supercomplexes, 1.5‐NR is a subset of 1.5‐PSD95. Accordingly, we quantified that there is 17‐fold more 1.5‐PSD95 than GluN1. Because ~50% of GluN1 is in 1.5‐NR and ~50% in 0.8‐NR (ion channel complexes alone; lacking PSD95) (Frank *et al*. 2016) then 1/34 of all PSD95 supercomplexes contain NMDAR in mouse forebrain (Fig. 3g). These molar ratios were from total forebrain and differ somewhat from estimates using mass spectrometric approaches on Triton X‐100‐resistant fractions of forebrain membranes (Cheng *et al*. 2006). It is likely each ~1.5 MDa supercomplex can accommodate a single tetrameric NMDAR. Since TAP‐purified NMDARs contain approximately equal amounts of PSD95 and PSD93 (Frank *et al*. 2016) and the apparent dimeric oligomeric state of PSD95 *in vivo*, we suggest 1.5‐NR supercomplexes are organized around a core platform containing a dimer of PSD95 and a dimer of PSD93 (Hsueh *et al*. 1997).

Supercomplexes containing PSD95 with or without NMDARs are further subdivided into 1.5‐Arc, 1.5‐IQsec2, 1.5‐Kir2.3 and 1.5‐Adam22 supercomplex subfamilies. This is consistent with the high degree of overlap of proteins identified by mass spectrometry samples purified from *Grin1* ^*TAP*^ and *Psd95* ^*TAP*^ mice (Frank *et al*. 2016). While the similarity between these proteomes might suggest that synaptic proteins associate promiscuously or by redundant mechanisms, we show using several mouse mutations that some supercomplexes have strict and selective genetic dependencies for the assembly of their constituent proteins. Interestingly, a similar genetic mechanism has been shown to organize ankyrins, a family of axonal scaffold protein, that cluster ion channels at the nodes of Ranvier (Ho *et al*. 2014).

The characterization of the Kir2.3‐NMDAR ion channel‐channel supercomplexes and their neuroanatomical distribution highlights the potential physiological importance of mechanisms controlling the organization of supercomplexes. A functional interaction between inward‐rectifying potassium channels and NMDARs has been predicted to be the ‘perfect couple’ for producing the necessary voltage bi‐stability of ‘on’ and ‘off’ states within the postsynaptic membrane (Major *et al*. 2013; Sanders *et al*. 2013). Our finding that a subset of 1.5‐Kir2.3 supercomplexes required the cytoplasmic domain of GluN2B suggests that this domain may be directly involved with the mechanism of supercomplex formation between these two channels. Kir2.3‐NMDAR ion channel‐channel supercomplexes were anatomically enriched within the ventral midbrain regions but absent from the hippocampus and cortex. Thus, the mechanisms regulating the hierarchical organization of synaptic supercomplex subfamilies appear to specify the diversity of synapse subtypes.

Our results also shed light on the molecular organization of synapses in vertebrate organisms and how their supercomplex diversity arose. Two whole genome duplications occurred ~550 million years ago in the vertebrate lineage resulting in the generation of paralogs and an overall expansion of the vertebrate synapse proteome (Emes *et al*. 2008; Emes and Grant 2012). Our present results show selective effects of paralog mutations in PSD95 and PSD93, as well as GluN2A and GluN2B. Thus, the increase in paralogs has not simply multiplied the number of vertebrate supercomplexes, but rather, the diversification of paralogs has resulted in selective functions that restrict the diversity of supercomplexes.

The hierarchical organization of supercomplexes outlined here is potentially generally relevant to the 66 proteins we have previously reported to be distributed between 220 separable synaptic complexes and supercomplexes (Frank *et al*. 2016). Indeed, it is possible that the number of distinct complexes and supercomplexes identified using this biochemical approach is underestimated because weakly associated or very low abundance constituents may be refractory to the use of detergents. We propose the hierarchy of genetic requirements that gives rise to the assembly of distinct supercomplex subfamilies may play an important role in defining synaptic function and synaptic subtypes of the brain. Given the diversity of complexes and organization into subfamilies, we suggest there is potential to define a taxonomy based on composition and its genetic determinants.
